# Clinical utility of liquid biopsy-based companion diagnostics in the non-small-cell lung cancer treatment

**DOI:** 10.37349/etat.2022.00104

**Published:** 2022-10-31

**Authors:** Yoshiharu Sato

**Affiliations:** DNA Chip Research Inc., Tokyo 105-0022, Japan; University of Campania “Luigi Vanvitelli”, Italy

**Keywords:** Liquid biopsy, companion diagnostics, genome informatics

## Abstract

Recently, technological advances in the detection and biological characterization of circulating tumor DNA (ctDNA) have enabled the implementation of liquid biopsy testing into clinical practice. Methods for analysis of liquid biopsies have rapidly evolved over the past few years and have continued to advance, thus providing details about tumor biological characteristics such as tumor progression, metastasis, tumor heterogeneity, genomic mutation profile, clonal evolution, etc. In tandem with technological advances, the implementation of liquid biopsy in routine clinical settings has proceeded. In 2016, the Food and Drug Administration (FDA) approved the first ctDNA liquid biopsy test to detect epidermal growth factor receptor (*EGFR*) gene mutations in patients with non-small-cell lung cancer (NSCLC) as a companion diagnostic for molecular targeted drug of EGFR-tyrosine kinase inhibitor (TKI, EGFR-TKI). More recently, multigene panel assays of liquid biopsy have been approved as companion diagnostics and have been used in routine clinical settings. The estimation of blood tumor mutation burden (bTMB) to predict the efficacy of immune checkpoint inhibitor (ICI) treatment can be one of the promising approaches to liquid biopsy. The next stage of implementation of liquid biopsy for routine clinical settings is for monitoring of ctDNA after surgical treatment to predict prognosis and to detect disease relapse earlier than conventional imaging diagnosis. Its clinical utility is under assessment in several clinical trials. This review introduces recent advances in liquid biopsy methodology, the development of biomarkers, and its clinical utility in the treatment of NSCLC patients.

## Introduction

Lung cancer is the leading source of cancer-related deaths around the world, with more than 1.8 million deaths in 2020 [1]. In recent decades, molecular target drugs have been approved for epidermal growth factor receptor (*EGFR*), anaplastic lymphoma kinase (*ALK*), ROS proto-oncogene 1, receptor tyrosine kinase (*ROS1*), MET proto-oncogene, receptor tyrosine kinase (*MET*), B-Raf proto-oncogene, serine/threonine kinase (*BRAF*), rearranged during transfection (*RET*), kirsten rat sarcoma viral oncogene homologue (*KRAS*), and neurotrophic tyrosine receptor kinase (*NTRK*) mutations. The advance in the development of molecular targeted drugs accelerates the precision medicine/tailer-made medicine of non-small-cell lung cancer (NSCLC) patients, which greatly improves the quality of life, and prolongs survival of NSCLC patients [2–4]. Molecular targeted drugs have been developed continuously and the covered fraction of driver mutations among NSCLC patient populations will increase [e.g., *EGFR* exon 20 insertions, neuregulin 1 (*NRG1*), human epidermal growth factor receptor 2 (*HER2*), etc.]. The enrichment of druggable driver mutations with available companion diagnostics (CDx) increases the importance of multiple panel testing. For reliable and proficient panel testing, tissue-based next-generation sequencing (NGS) panel assay such as Oncomine Dx Target Test (DxTT) [5] or FoundationOne CDx [6] is basically prioritized over minimally invasive liquid biopsy panel testing due to inferiority of sensitivity, since circulating tumor DNA (ctDNA) is not always sufficiently flowed in the bloodstream for a not-negligible substantial fraction of patient populations. The bulk of cell-free DNA (cfDNA) derives from apoptotic hematopoietic cells, while in cancer patients, it includes ctDNA. Liquid biopsy has many practical advantages and is considered a favorable option when tissue biopsy specimens are not available or scant, or when invasive tissue biopsy is not desirable (e.g., in the settings of re-biopsy, or high fibrillization). In recent years, several liquid biopsy CDx have been approved by Food and Drug Administration (FDA) as shown in Table 1.

**Table 1.** FDA-approved liquid biopsy CDx testing for NSCLC treatment

**Name of test**	**Mutation**	**Drugs**	**Technology**	**Limit of detection**
Cobas EGFR mutation test v2	EGFR exon 19 deletion EGFR L858R EGFR T790M EGFR uncommon mutations	Gefitinib, erlotinib, osimertinib	RT-PCR	5% [7]
Gurdant 360 CDx	EGFR mutations EGFR exon 20 insertion KRAS Gly12Cys	Osimertinib, amivantamab, sotorasib	NGS panel (61 genes)	0.25% [8] 1.50% (package insert)
FoundationOne Liquid CDx	EGFR mutations (exon 19 deletion, L858R) ALK fusion MET exon 14 skipping	Gefitinib, erlotinib, osimertinib, alectinib, capmatinib	NGS panel (318 genes)	0.27∼0.94% [9]
ArcherMET	MET exon 14 skipping	Tepotinib	NGS	0.20% (package insert) [10]

RT-PCR: reverse transcription polymerase chain reaction

Druggable driver mutations now cover more than 50% of the NSCLC patients [11], however, treatment options other than molecular targeting drugs have been desired for the remaining half of the populations (driver mutation-negative). Immune checkpoint inhibitor (ICI) is the therapeutic agent which blocks the immune checkpoint system and unlocks the immune evasion of the tumor, resulting in the restoration of tumor recognition by the host immune system. ICI can be used as one of the treatment options for driver mutation-negative NSCLC patient populations [12]. However, the efficacy of ICI is limited to only 20% of patients without any selection [13–15], thus increasing the need of predictive biomarkers for ICI efficacy. Tumor mutation burden (TMB) from plasma ctDNA by liquid biopsy is one of the promising biomarkers for ICI efficacy prediction [16]. Furthermore, one of the utilization of liquid biopsy is the measurement of minimal residual disease (MRD) as the biomarker for adjuvant therapy or early detection of recurrent disease. A recent study suggested that EGFR-tyrosine kinase inhibitor (TKI, EGFR-TKI) adjuvant therapy for resectable NSCLC patients showed great improvement in the treatment outcome [prolong the disease-free survival (DFS)] [17]. Currently, many research studies on MRD detection have been conducted to explore the predictive biomarker to discriminate patients with a high risk of recurrence from patients with complete eradication.

This review will focus on the clinical utility of liquid biopsy, especially for CDx in the treatment of NSCLC patients. Moreover, this review mainly will focus on the ctDNA-based approach among possible liquid-based biomarkers, because most of the current technologies of liquid biopsy for CDx implemented in clinical settings are ctDNA-based genomic profiling. In this review, the papers published from 2010 to 2022 were analyzed.

## Clinical utility of ctDNA dynamics in the treatment of NSCLC

The ctDNA dynamics can be evaluated by monitoring changes in ctDNA level in patient follow-up, which is one of the promising biomarkers of liquid biopsy. Many studies of liquid biopsy monitoring and quantifying ctDNA have been conducted to establish a novel approach for prediction of treatment response and monitoring of disease progression, and prediction of prognosis. In the study by Taus et al. [18], the EGFR mutation status of 221 plasma samples from 33 lung cancer patients was analyzed by droplet digital polymerase chain reaction (ddPCR) or beads, emulsions, amplification, magnetics (BEAMing). In the retrospective study, the authors assessed the association of *EGFR* mutations in plasma ctDNA with the radiologic response [18]. The authors reported that *EGFR* mutation allele frequency of plasma ctDNA predicted treatment response of EGFR-TKI and disease progression in advance of radiological evaluation in 93% and 89% of cases, respectively. In the ALEX study, the research group retrospectively assessed the prognostic value of plasma cfDNA for 276 NSCLC patients with *ALK* mutation positive. The association between cfDNA quantity in plasma and patient outcomes was analyzed using a Cox regression model. The median cfDNA concentration from plasma of 276 patients was 11.53 ng per 1 mL plasma. The concentration of cfDNA was revealed to positively correlate with tumor size (tumor burden), the number of lesions, and the number of organ lesion sites. The patients in the high cfDNA amount group (cutoff was determined at the upper 50 percentile point from the study population) were more likely to experience disease progression than the patients in the low cfDNA amount group [19]. In the study by Knapp et al. [20], they evaluated the feasibility of ctDNA testing on a commercial fixed-gene panel (InVisionFirst^®^-Lung to detect mutations in 36 cancer-related genes) to predict the recurrence risk of chemoradiation therapy (CRT) in patients with locally advanced NSCLC. Plasma samples from 43 patients were collected at CRT initiation, CRT completion, and quarterly follow-up for 12 months. Progression-free survival (PFS) was longer in patients with diminished ctDNA compared to those with detectable ctDNA (median PFS 567 days *vs.* 74 days). The study by Palmero et al. [21] prospectively assessed the NGS panel assay of ctDNA compared with standard-of-care (SoC) tissue-biopsy testing to identify guideline-recommended actionable mutations in NSCLC. In total, 186 patients were enrolled in the study. The primary objective was noninferiority of ctDNA testing against tissue-based analysis for the detection of eight actionable biomarkers (including guideline-recommended *EGFR* and *ALK*). The non-inferiority of ctDNA testing was confirmed by the identification of actionable mutations in 46 patients by liquid *vs.* 48 by tissue. Druggable/actionable alterations or *KRAS* mutations were identified in 80.7% by liquid first testing *vs.* 57.1% by tissue first testing. Positive predictive value (PPV) for EGFR mutation detection on ctDNA was 100.0% (25/25). The study by Schouten et al. [22] assessed the additional clinical utility of ctDNA compared with SoC tissue-based molecular profiling (TMP) in the primary diagnostic setting of NSCLC in the Netherlands. In the study, pretreatment plasma samples from 209 NSCLC patients were analyzed retrospectively using the NGS AVENIO ctDNA 17 genes panel assay. SoC-TMP alone detected actionable drivers in 34.4% of patients. Plasma-based cfDNA-based molecular profiling (CMP) increased this detection rate to 39.7%. For potentially targetable drivers, the concordance of mutation calls between SoC-TMP and plasma-CMP was 86.6%. They concluded that plasma-CMP is a promising tool in CDx, although it cannot fully replace tissue-based mutation profiling by panel testing. In the study by Fiala et al. [23], the association between ctDNA response and treatment outcome of chemotherapy was validated. Eighty-four patients were enrolled in this study. The authors reported that the combination of quantitative ctDNA dynamics with positron emission tomography/computed tomography [PET/CT, changes in the maximum standardized uptake value (SUV_max_)] was promising for the prediction of objective response rate and PFS. The research group of Jiaotong University in China conducted a large cohort study of liquid biopsy investigating 5,000 Chinese patients with lung cancer. In the study, 5,671 blood samples from 4,892 patients with lung cancer were analyzed by a 1,021-gene panel. They also analyzed the concordance of mutation calls between matched tissue and germline white blood cells. The authors suggested that the evolutionary clonality pattern and co-mutation status with *EGFR* mutations could predict the treatment efficacy of EGFR-TKI. Furthermore, loss-of-function mutations of tumor suppressor gene retinoblastoma 1 (*RB1*) in NSCLC patients correlated with the subset of high blood TMB (bTMB), elevated plasma ctDNA load, and potential small cell transformation. The authors also established the optimal bTMB threshold to predict ICI efficacy for patients with extremely low ctDNA levels [24]. In the cohort study by Cheng et al. [25], plasma samples from 43 patients in the imaging cohort were collected before treatment initiation and serially before each cycle of therapy, and major driver mutations in ctDNA were analyzed by ddPCR. The detection of plasma ctDNA preceded the radiographic detection by a median of 24 weeks. These quick response characteristics of ctDNA may offer an early insight for treatment response in advance of the detection by the current SoC imaging approach.

## Application of liquid biopsy for the predictive biomarker of ICI efficacy

The response rate of ICI, in general, is only 15∼20% in the treatment of unselected NSCLC. Moreover, during the first few weeks, a substantial fraction of patients demonstrate radiological progression even if the ICI treatment is effective, which is called a pseudo-progression problem. This pseudo-progression problem harbors the difficulty of the assessment of ICI response in the early period after initial drug therapy. Until now, programmed cell death ligand 1 (*PD-L1*) expression, microsatellite instability (MSI), TMB, and circulating tumor cells (CTCs) have been widely investigated as potential predictive biomarkers for ICI efficacy. *PD-L1* expression has been implemented as a CDx biomarker in clinical settings. The performance of *PD-L1* biomarkers shows different results among clinical trials due to the utilization of different methods/antibodies among platforms/drugs, implementation of different cutoffs, and insufficient standardization of diagnostics as summarized in Table 2. TMB was also investigated as a promising predictive biomarker of ICI treatment. In principle, higher TMB has been assumed to show a better response rate of ICI, since high TMB may induce host immunity by the neo-antigen presentation. In fact, several pre-clinical/clinical trials showed that patients with high TMB tend to show better treatment responses than those with low TMB [26–28]. The research group of Memorial Sloan Kettering Cancer Center conducted a retrospective cohort study to explore the predictive biomarker of ICI treatment by investigating 1,714 cancer patients from 16 tumor types. The authors reported that a high neutrophil-to-lymphocyte ratio (NLR) correlated with poorer ICI response, overall survival, and PFS. Furthermore, the predictive performance was improved by integrating the TMB score with the NLR index. The cutoff of NLR was determined from the top 20 percentile points among whole participants in the study. The response rate of ICI was 28.8% for NLR low group and 18.1% for NLR high group. The response rate of the “TMB high/NLR low” group was increased to 38.2%, suggesting that the combination of TMB and NLR enables further proficient stratification. The cost-effectiveness and routine implementation of blood cell count in the current clinical settings is also advantage of NLR.

**Table 2.** FDA-approved anti-PD-L1 drugs and CDx for NSCLC treatment

**Drugs**	**Anti-PD-L1 antibody**	**Stratifiation (biomarker scoring)**	**Fraction of biomarker positive**	**Clinical trial**
Pembrorizumab	22C3-pharmDx (Dako)	TPS < 1%: PD-L1 negative 1% ≤ TPS < 50%: PD-L1 positive TPS ≥ 50%: PD-L1 positive	1% ≤ TPS < 50%: 37.6% TPS ≥ 50%: 23.2%	KEYNOTE-001 [29]
Nivolumab	28-8-pharmDx (Dako)	Expression < 1%: PD-L1 negative Expression ≥ 1%: PD-L1 positive	Expression ≥ 1%: 45.6%	CheckMate 227 [30]
Atezolizumab	SP142 (VENTANA)	TC, IC < 1%: PD-L1 negative TC or IC ≥ 1%: PD-L1 positive	TC or IC ≥ 1%: 54.9%	OAK [31]

TPS: tumor proportion score (fraction of PD-L1 expressing tumor cells); TC: fraction of PD-L1 expressing cells in the total tumor cells; IC: fraction of PD-L1 expressing cells in tumor infiltrating immune cells; OAK: atezolizumab *vs.* docetaxel in patients with previously treated non-small-cell lung cancer

Mismatch repair (MMR) status is also a promising predictive biomarker of ICI response [32–34]. The impact of MMR status as a predictive biomarker of ICI response has been limited since MSI high/MMR deficiency (dMMR) was found for only 0.17∼0.21% of NSCLC patients [35, 36]. Since none of the currently established biomarkers reaches sufficient predictive performance, many research studies to explore emerging biomarkers have been conducted in recent years. In the study by Shi et al. [24], the authors conducted a study investigating 637 Chinese NSCLC patients with ICI treatment to explore novel biomarkers for ICI responses. In addition to the TMB and PD-L1 expression, the mutation status of the tumor-suppressor gene tumor protein p53 (*TP53*) and lysine methyltransferase 2C (*KMT2C*) contributed to the prediction of ICI response. The patients with *TP53*/*KMT2C* co-mutation status showed longer PFS than patients with wild-type status. Brown et al. [37] reported that loss-of-function mutation of the low-density lipoprotein receptor-related protein 1B (*LRP1B*) tumor suppressor gene was a predictive biomarker of ICI response. In the study, 101 patients with ICI treatment and with available *LRP1B* mutation information were investigated. The overall response rate of ICI for patients with *LRP1B* mutation showed 54%, which was a better response than that of the patient with variant of unknown significance (VUS) alterations (13%).

CTC is another potential biomarker for ICI response prediction. Similar to the detection of PD-L1 expression levels, CTC also gives us useful information about tumor characteristics. In contrast to ctDNA-based profiling, the information on RNA/protein expression of tumor cells can be obtained by liquid biopsy-based CTC analysis. Single-cell analysis of CTCs may be a promising approach to analyzing the intra-tumor and inter-tumor heterogeneity. By single cell analysis of CTCs, we can assess the evolutionary scenario and sub-clonality pattern/populations of the tumor microenvironment, which may contribute to the detailed profiling of tumor status and to ICI efficacy prediction. Further investigation of the clinical utility of CTC and cost reduction of CTC analysis will be needed for future clinical implementation.

As other biomarkers in the category of genomic mutation status, phosphatase and tensin homolog (*PTEN*) (tumor suppressor), DNA polymerase epsilon catalytic subunit (*POLE*) (a critical protein involved in DNA proofreading and replication), *KRAS*-serine/threonine kinase 11 (*STK11*) (tumor suppressor) co-mutation status have shown promising predictive performance. In the category of emerging technology, the status of tertiary lymphoid structure (TLS) of the tumor microenvironment is a promising biomarker candidate. Recently, several findings of independent studies indicate that B cells infiltrating into the tumor tissue and TLSs are key determinants of response to ICI. The enrichment/cluster of B-cells linked with localization of switched memory B-cell was assumed to correlate with better ICI efficacy, suggesting the feasibility of TLS analysis as ICI response prediction [38, 39]. The integrative assessment of TLS status by imaging and less invasive liquid biopsy testing to analyze B-cell repertoire may be a promising approach for ICI efficacy prediction.

In the phase III MYSTIC trial, bTMB and tissue TMB (tTMB) were assessed using GuardantOMNI (2.145 Mb size panel) and FoundationOne CDx assays, respectively. In the study, the optimal bTMB cutoff was analyzed by the minimal *P* value cross-validation approach and Cox proportional hazards model. The authors confirmed that the success rate to obtain an optimal TMB score was higher for bTMB than for tTMB, suggesting the advantage of blood-based TMB count. They found the optimal bTMB cutoff of 20 mutations/Mb or higher to predict durvalumab + tremelimumab efficacy. High bTMB was predictive of clinical benefit by ICI treatment *vs.* chemotherapy [40]. In the study by Wang et al. [41], bTMB was estimated by NCC-GP150 (150 genes panel) and the association of bTMB with PFS of anti-PD-1/anti-PD-L1 treatment was evaluated. In the study, matched tissue and plasma samples from 48 patients with advanced NSCLC were analyzed. In the cohort, the authors confirmed that a bTMB of 6 or higher was associated with significantly longer PFS [hazard ratio (HR), 0.39; 95% confidence interval (CI), 0.18–0.84]. High bTMB group also showed superior objective response rates (bTMB ≥ 6: 39.3%; 95% CI, 23.9–56.5; bTMB < 6: 9.1%; 95% CI, 1.6–25.9). Nabet et al. [42] reported that the combination of the pre-treatment ctDNA level and peripheral CD8 T cell levels associate with durable clinical benefit (DCB) for NSCLC patients receiving ICIs. In the study by Weber et al. [43], the molecular response of ctDNA after ICI treatment was validated as a predictor of tumor progression and long-term survival benefits of ICI treatment. The authors confirmed that STK11/kelch like ECH associated protein 1 (KEAP1) dual mutation and ctDNA levels (HR, 2.08; 95% CI, 1.4–3.0) were independent predictors for overall survival, irrespective of PD-L1 expression status [44]. As another emerging biomarker, the co-mutation of DNA damage response (DDR) pathways has been reported to associate with improved survival for ICI treatment of NSCLC patients [44]. In the study, a total of 853 NSCLC patients were analyzed to assess the association between co-mutation status and clinical outcomes with atezolizumab treatment. Co-mutation-positive patients showed significantly prolonged PFS and overall survival (OS) for the treatment of atezolizumab *vs.* chemotherapy. Co-mutation status still predicted improved clinical benefit from atezolizumab therapy for patients with low or negative PD-L1 expression. In the study by Lu et al. [45], the authors confirmed the association between ICI treatment response and mutation status of ubiquitin-like conjugation (UBL) biological process genes. The UBL genes included ABL proto-oncogene 1 (*ABL1*), activated protein C (*APC*), low-density lipoprotein receptor-related protein 6 (*LRP6*), far upstream element binding protein 1 (*FUBP1*), *KEAP1*, and DNA topoisomerase II alpha (*TOP2A*). In the study, the ctDNA samples from 399 patients were analyzed by molecular profiling panel assay. The patients with UBL mutation (UBL^+^) had shorter PFS (1.69 months *vs.* 3.22 months) and shorter OS (8.61 months *vs.* 16.10 months) than those patients with wild-type UBL (UBL^–^) [45]. Since the proteasomal degradation process is the important biological pathway in the antigen presentation and innate/adaptive immunity process, the shorter PFS for the patients with aberrant UBL systems agrees with the biological speculation.

## Minimally invasive CDx using cytology/liquid samples

Minimally invasive CDx by liquid biopsy is expected to reduce the burden on the patient. However, from the viewpoint of the diagnosis success rate and detection rate of driver mutation, liquid biopsy panel testing can not be used as a complete replacement for tissue-based panel testing. The main hurdle for this can be explained by the fact that a sufficient amount of ctDNAs are not always effused into the patient’s bloodstream. The effusion rates of ctDNA are known to be different among patients, stages, and cancer types [46]. Continuous formalin-fixed paraffin-embedded (FFPE) slides and confirmation of tumor cells by hematoxylin and eosin stain (H&E stain) in the adjacent slide is the common procedure to guarantee the existence of a sufficient amount of neoplastic cells in the initial nucleotide material for the panel assay. However, tissue-biopsy is not available, or difficult to obtain a sufficient amount of tissue specimens for a certain fraction of patients, and doctors may avoid invasive tissue biopsy in some situations. In these cases, minimally invasive liquid biopsy is the backup option of multi-gene panel assay for CDx. For this purpose, a cytology specimen is one of the options of backup materials for CDx. Studies examining backup materials for molecular testing in NSCLC treatment are listed in Table 3. Huber et al. [47] used sputum specimens for mutation analysis and successfully detected EGFR mutation. In the study, sputum DNA from 10 lung cancer patients with *EGFR* mutation (confirmed by tissue specimen) was analyzed by cycleave polymerase chain reaction (PCR), targeted resequencing, and ddPCR. EGFR mutations were detected in maximally 50% of the sputum samples of patients with EGFR mutations. Morikawa et al. [48] reported a successful multigene NGS panel implementing 8 druggable genes, using sputum specimens from 3 NSCLC patients. They successfully detected druggable mutations from all three patients (case1: *MET* skipping, case2: *EGFR* exon 19 deletions, case3: *KRAS* Gly12Ala) [48]. Bronchial washing fluid (BWF) is another promising minimally invasive liquid biopsy material for mutation panel assay. In several studies, liquid biopsy panel assays were performed using BWFs and authors reported that they obtained substantially concordant results with tissue-based panel testing [49–51]. In the study by Roncarati et al. [49], 73 lung cancer patients were analyzed. Concordance with gene mutations uncovered in tumor tissue biopsies was higher than 90%. In the study by Zhai et al. [50], concordance of mutation calls among matched tumor tissues with a soluble fraction of BWF (BWFs), a precipitate fraction of BWF (BWFp), and pre-treatment plasma samples were validated for 19 patients. For these patients, 204 somatic mutation variants were detected in tissue samples. As a result, 189 (92.6%), 175 (85.5%), and 163 (79.9%) mutations were detected in the matched BWFs, BWFp, and plasma samples, respectively. These results suggest the usefulness of BWFs as a minimally invasive liquid biopsy for CDx gene panel assay. In the study by Tu et al. [52], they analyzed 164 matched tumor tissues and ctDNA from pleural effusion of advanced lung cancer patients. They assessed the concordance of mutation call results from 153 matched plasma samples and from 63 pleural effusion-derived sediment DNA (PE-sDNA, DNA purification from cell sediment of pleural effusion) samples using panel results from tissue specimens as gold-standard reference. The cell-free fraction of pleural effusion (PE-cfDNA) displayed significantly higher variant allele frequency than that of plasma cfDNA. Furthermore, a high concordance rate of 65% with tissue-based profiling was obtained for PE-cfDNA, which was higher than that of PE-sDNA (43%) and that of plasma-cfDNA (43%) [52]. From this study, cfDNA fraction of pleural effusion is assumed to be superior to the cell sediment fraction of pleural effusion for mutation profile. It is interesting to see the performance of pleural effusion material to detect driver mutations when the combined fractions of cell sediment fraction and cfDNA fractions are used for highly sensitive liquid biopsy panel testing.

**Table 3.** Assessment of the feasibility of non-plasma type biological specimens/materials for minimally invasive liquid biopsy

**References**	**Material**	**Method**	**The number of samples analyzed**	**Panel size (the number of genes)**
Hubers et al. [47]	Sputum	ddPCR, NGS panel	10	1 (EGFR)
Morikawa et al. [48]	Sputum	NGS panel	3	8 genes
Roncarati et al. [49]	Bronchiral washing fluids	NGS panel	73	14 genes
Zhai et al. [50]	Bronchiral washing fluids	NGS panel	20	1,021 genes
Zhang et al. [51]	Bronchiral washing fluids	PCR (Alldetect™ EGFR Mutation Test Kit)	144	1 (EGFR)
Tu et al. [52]	Pleural effusion	NGS panel	32	448 genes

## MRD monitoring in the setting of adjuvant molecular target therapy for resectable NSCLC

Recently, the benefit of adjuvant EGFR-TKI therapy for the treatment of resectable NSCLC has been investigated and positive results have been reported. The results of the ADAURA trial investigating the clinical utility of osimertinib adjuvant therapy for resectable NSCLC have been reported [20, 21]. In the study, 682 NSCLC patients were enrolled, and a randomized trial of osimertinib adjuvant treatment group and placebo group was conducted. The primary endpoint was DFS among patients with stages II to IIIA disease. At 24 months, 90% of the patients in osimertinib group were alive and disease-free, compared with 44% of patients in the placebo group. The authors concluded that DFS was significantly longer among patients in osimertinib group than among those in the placebo group. Furthermore, the subsequent study supported the adjuvant osimertinib as an effective treatment for resectable NSCLC regardless of the previous history of adjuvant chemotherapy [22]. Although the result of the ADAURA trial is very promising, the fact that a substantial fraction of patients may be cured by surgery alone poses the question of additional toxic adjuvant therapy for all EGFR mutation-positive patients. Therefore, the biomarker is needed to screen patients who can get benefit from adjuvant EGFR-TKI therapy. MRD is a remaining tumor-derived material, which can be detected in the bloodstream of the patient even after surgical resection of the tumor. Therefore, MRD can be measured by liquid biopsy quantifying ctDNA. MRD has been considered associated with a poor prognosis of resectable NSCLC. From the viewpoint of medical economy and precision medicine, new biomarkers such as MRD monitoring and molecular profiling for ctDNA may be integrated in addition to the EGFR mutation status to effectively stratify patients with a high risk of recurrence from patients with a low risk of recurrence. In the ADJUVANT study, authors reported the comparative superiority of adjuvant gefitinib over chemotherapy in DFS of resected *EGFR*-mutant stage II–IIIA NSCLC [23]. In this trial, five predictive biomarkers from genomic mutational profiles were identified. These mutational biomarkers were *TP53* mutations, *RB1* mutations, and copy number amplifications of Nkx homeobox-1 gene (*NKX2-1*) (essential gene for early lung morphogenesis), cyclin dependent kinase 4 (*CDK4*) (protein-serine kinase involved in the cell cycle), and *MYC* (proto-oncogene and encodes a nuclear phosphoprotein that plays a role in cell cycle progression, apoptosis, and cellular transformation). Furthermore, patients were categorized into three subgroups with relative DFS and overall survival benefits from either adjuvant gefitinib or chemotherapy (“TKI is highly preferable”, “TKI is preferable”, and “Chemotherapy is preferable” groups). The predictive genomic signatures showed promising potential as predictive biomarkers for personalized medicine of molecular targeted adjuvant therapy. The study suggested that integrated assessment of gene signature scores from tissue-based mutation profiling and ctDNA MRD may be promising biomarkers for adjuvant therapy. In the multicenter cohort on dynamic monitoring of ctDNA in lung cancer surgery patients (LUNGCA), the clinical utility of MRD was investigated. A total of 330 NSCLC patients with stages I–III were enrolled. Plasma samples were collected at 3 time points (before surgery, 3 days, and 1 month after surgery) and purified plasma cfDNA samples were subjected to 769 genes custom panel assay. The patients in the group with detectable ctDNA showed significantly shorter recurrence-free survival (RFS) than those in the group with ctDNA negative. The MRD detection at postoperative 3 days and/or 1 month was a strong predictor for disease relapse. They suggested that receiving adjuvant therapies was an independent predictive factor for RFS in the MRD-positive population but not in the MRD-negative population [24]. Chaudhuri et al. [53] investigated 255 plasma samples from 40 patients treated with curative therapy for stage I–III lung cancer and 54 healthy adults. In the study, the detection of ctDNA during the postoperative period preceded radiographic detection in 72% of patients by a median lead time of 5.2 months. The early detection of MRD enables treatment intervention at early time points when the disease burden is lowest. In the study by Qiu et al. [54], ctDNA detection precedes radiological recurrence detection by a median of 88 days. Using joint modeling of longitudinal ctDNA analysis and time-to-recurrence, they accurately predict postsurgical recurrence status [53]. In the study by Gale et al. [55], they analyzed 363 plasma samples from 88 patients with early-stage NSCLC (48.9%, 28.4%, and 22.7% at stages I, II, and III, respectively). Plasma ctDNA was detected for 17% of patients within the timepoint of 2 weeks to 4 months. Patients with detectable ctDNA at this timepoint showed significantly shorter RFS (HR, 14.8) and overall survival (HR, 5.48) than those with ctDNA negative. The detection of ctDNA at 1–3 days after treatment showed no association with the recurrence of the tumor. Detection of ctDNA before treatment was associated with shorter overall survival and RFS [54].

## Conclusions

The clinical utility of liquid biopsy was evidenced by the implementation of liquid biopsy CDx in routine clinical settings. Several studies have reported that TMB estimation by liquid biopsy was useful for ICI response prediction. In addition to the PD-L1 expression, integration and combinatorial scoring of TMB, MSI, NLR, and genomic mutation profiles improved the performance of ICI efficacy prediction. However, the performance seems not to reach a sufficient level, thus further improvement will be continued in this research field. From the viewpoint of medical economics, cost-effectiveness will be another important factor in the development of biomarkers. The combinatorial and integrative approach of multi-omics/multi-platform diagnostics may pose an increase in analytical cost and difficulty in standardization among facilities. Increase of druggable mutations with available molecular targeted drugs in the NSCLC treatment altered the procedure of CDx. Sequential and prioritized testing by single-plex CDx has been replaced by an all-in-one multi-panel CDx assay from the viewpoint of cost-effectiveness and the limitation of specimens. Further increase of druggable mutations may enable the routine clinical use of large panel testing or comprehensive analysis for 1st line CDx, in which TMB or genetic profiling results can be incorporated to improve the performance of personalized medicine at early timing of treatment.

Minimally invasive CDx by liquid biopsy will further extend its application. Shorter turn around time (TAT) of liquid biopsy is also the practical merit of liquid-based CDx, thus providing options for several situations (e.g., urgent cases). Improvement of highly accurate panel assay such as the implementation of the molecular barcoding method [8, 55] enables us to detect ctDNA with extremely high specificity and sensitivity. The successive cost down of NGS will further enhance the implementation of liquid biopsy in routine clinical practice. The high specificity of liquid biopsy testing and minimally invasiveness may provide the benefit of the first screening of druggable mutations at an early time point (e.g., liquid biopsy CDx at the time of first medical examination).

The complexity of medical treatment sequences has increased due to the emergence of various modalities and the establishment of new treatments and biomarkers. The clinical utility of liquid biopsy-based CDx by ctDNA molecular profiling has been proved by the fact that several CDx have continued to be approved and implemented in clinical settings such as Cobas for EGFR, ArcherMET for MET exon 14 skipping, and Guardant 360 CDx for KRAS G12C. Poorer sensitivity for the detection of RNA fusion by liquid biopsy compared to tissue-based CDx is the limitation of liquid biopsy-based CDx. Liquid biopsy-based CDx will be applied and expanded to other tumor types, in which tissue biopsy is difficult and multiple druggable mutations are available. As the alternative less invasive liquid biopsy-based CDx, cytology samples will be promising backup material for molecular testing, considering the good balance of analytical performance (sensitivity and specificity) and invasiveness compared with liquid biopsy by plasma samples. Re-biopsy CDx panel testing at the timing of 2nd line treatment will be effective to support treatment decisions based on the profiling of resistant mutations from specimens at the timing of progressive disease (PD). This has been supported by the recent findings that druggable mutations were detected for a substantial population at the timing of PD of 1st line EGFR-TKI treatment. Performing of tissue re-biopsy at PD is technically challenging because of many hurdles. Therefore, panel testing using cytology samples will be effective for precision medicine at this time of CDx. Several studies have shown the usefulness of MRD quantification as a biomarker of adjuvant ICI/TKI therapy for resectable NSCLC and other tumor types. Although several studies have suggested promising features and good performance of MRD monitoring, further validation of clinical utility by prospective clinical trials will be required for the wide use of MRD monitoring in routine clinical settings. bTMB showed moderate improvement in predictive power as a biomarker for ICI therapy compared with PD-L1 expression. However, the predictive power of bTMB alone is limited. Therefore, further exploration of a novel biomarker or combination formula of integrative use of multiple biomarkers will be needed for the complete replacement of SoC testing, considering the balance of performance and cost-benefit.

How the difference in treatment sequence contributes to the benefit of the patient is the key question. In the future, the clinical utility of liquid biopsy with emerging methodology will be assessed by a well-designed prospective study.

## Abbreviations

ALK: anaplastic lymphoma kinase
bTMB: blood tumor mutation burden
BWF: bronchial washing fluid
CDx: companion diagnostics
cfDNA: cell-free DNA
CI: confidence interval
CMP: cell-free DNA-based molecular profiling
CRT: chemoradiation therapy
CTCs: circulating tumor cells
ctDNA: circulating tumour DNA
ddPCR: droplet digital polymerase chain reaction
DFS: disease-free survival
EGFR: epidermal growth factor receptor
FDA: Food and Drug Administration
HR: hazard ratio
ICI: immune checkpoint inhibitor
KRAS: kirsten rat sarcoma viral oncogene homologue
LRP1B: low-density lipoprotein receptor-related protein 1B
MET: MET proto-oncogene, receptor tyrosine kinase
MMR: mismatch repair
MRD: minimal residual disease
MSI: microsatellite instability
NGS: next-generation sequencing
NLR: neutrophil-to-lymphocyte ratio
NSCLC: non-small-cell lung cancer
PD: progressive disease
PD-L1: programmed cell death ligand 1
PFS: progression-free survival
RFS: recurrence-free survival
SoC: standard-of-care
TKI: tyrosine kinase inhibitor
TLS: tertiary lymphoid structure
TMB: tumor mutation burden
TMP: tissue-based molecular profiling
TP53: tumor protein p53
UBL: ubiquitin-like conjugation

## Declarations

### Copyright

© The Author(s) 2022.

## References

[1] Sung H Ferlay J Siegel RL Laversanne M Soerjomataram I Jemal A Global cancer statistics 2020: GLOBOCAN estimates of incidence and mortality worldwide for 36 cancers in 185 countries. CA Cancer J Clin. 2021;71:209–49. 10.3322/caac.21660 33538338

[2] Domvri K Zarogoulidis P Darwiche K Browning RF Li Q Turner JF Molecular targeted drugs and biomarkers in NSCLC, the evolving role of individualized therapy. J Cancer. 2013;4:736–54. 10.7150/jca.7734 24312144 PMC3842443

[3] Yang YP Ma YX Huang Y Zhao YY Fang WF Hong SD QoL analyses from INFORM study, a phase III study of gefitinib *versus* placebo as maintenance therapy in advanced NSCLC. Sci Rep. 2015;5:11934. 10.1038/srep11934 26137916 PMC4490396

[4] Chen G Feng J Zhou C Wu YL Liu XQ Wang C Quality of life (QoL) analyses from OPTIMAL (CTONG-0802), a phase III, randomised, open-label study of first-line erlotinib *versus* chemotherapy in patients with advanced EGFR mutation-positive non-small-cell lung cancer (NSCLC). Ann Oncol. 2013;24:1615–22. 10.1093/annonc/mdt012 23456778

[5] Lih CJ Harrington RD Sims DJ Harper KN Bouk CH Datta V Analytical validation of the next-generation sequencing assay for a nationwide signal-finding clinical trial: molecular analysis for therapy choice clinical trial. J Mol Diagn. 2017;19:313–27. 10.1016/j.jmoldx.2016.10.007 28188106 PMC5397672

[6] Milbury CA Creeden J Yip WK Smith DL Pattani V Maxwell K Clinical and analytical validation of FoundationOne^®^CDx, a comprehensive genomic profiling assay for solid tumors. PLoS One. 2022;17:e0264138. 10.1371/journal.pone.0264138 35294956 PMC8926248

[7] Benlloch S Botero ML Beltran-Alamillo J Mayo C Gimenez-Capitán A de Aguirre I Clinical validation of a PCR assay for the detection of *EGFR* mutations in non–small-cell lung cancer: retrospective testing of specimens from the EURTAC trial. PLoS One. 2014;9:e89518. 10.1371/journal.pone.0089518 24586842 PMC3934888

[8] Lanman RB Mortimer SA Zill OA Sebisanovic D Lopez R Blau S Analytical and clinical validation of a digital sequencing panel for quantitative, highly accurate evaluation of cell-free circulating tumor DNA. PLoS One. 2015;10:e0140712. 10.1371/journal.pone.0140712 26474073 PMC4608804

[9] Woodhouse R Li M Hughes J Delfosse D Skoletsky J Ma P Clinical and analytical validation of FoundationOne Liquid CDx, a novel 324-Gene cfDNA-based comprehensive genomic profiling assay for cancers of solid tumor origin. PLoS One. 2020;15:e0237802. 10.1371/journal.pone.0237802 32976510 PMC7518588

[10] Bormann Chung C Lee J Barritault M Bringuier PP Xu Z Huang WY Evaluating targeted next-generation sequencing assays and reference materials for NTRK fusion detection. J Mol Diagn. 2022;24:18–32. 10.1016/j.jmoldx.2021.09.008 34656759

[11] Fois SS Paliogiannis P Zinellu A Fois AG Cossu A Palmieri G. Molecular epidemiology of the main druggable genetic alterations in non-small cell lung cancer. Int J Mol Sci. 2021;22:612. 10.3390/ijms22020612 33435440 PMC7827915

[12] Calles A Riess JW Brahmer JR. Checkpoint blockade in lung cancer with driver mutation: choose the road wisely. Am Soc Clin Oncol Educ Book. 2020;40:372–84. 10.1200/EDBK_280795 32421452

[13] Borghaei H Paz-Ares L Horn L Spigel DR Steins M Ready NE Nivolumab *versus* docetaxel in advanced nonsquamous non-small-cell lung cancer. N Engl J Med. 2015;373:1627–39. 10.1056/NEJMoa1507643 26412456 PMC5705936

[14] Brahmer J Reckamp KL Baas P Crinò L Eberhardt WEE Poddubskaya E Nivolumab *versus* docetaxel in advanced squamous-cell non-small-cell lung cancer. N Engl J Med. 2015;373:123–35. 10.1056/NEJMoa1504627 26028407 PMC4681400

[15] Katayama Y Shimamoto T Yamada T Takeda T Yamada T Shiotsu S Retrospective efficacy analysis of immune checkpoint inhibitor rechallenge in patients with non-small cell lung cancer. J Clin Med. 2019;9:E102. 10.3390/jcm9010102 31906082 PMC7019787

[16] Gandara DR Paul SM Kowanetz M Schleifman E Zou W Li Y Blood-based tumor mutational burden as a predictor of clinical benefit in non-small-cell lung cancer patients treated with atezolizumab. Nat Med. 2018;24:1441–8. 10.1038/s41591-018-0134-3 30082870

[17] Wu YL Tsuboi M He J John T Grohe C Majem M et al.; ADAURA Investigators. Osimertinib in resected *EGFR*-mutated non-small-cell lung cancer. N Engl J Med. 2020;383:1711–23. 10.1056/NEJMoa2027071 32955177

[18] Taus Á Camacho L Rocha P Hardy-Werbin M Pijuan L Piquer G Dynamics of *EGFR* mutation load in plasma for prediction of treatment response and disease progression in patients with *EGFR*-mutant lung adenocarcinoma. Clin Lung Cancer. 2018;19:387–94.e233. 10.1016/j.cllc.2018.03.015 29656868

[19] Dziadziuszko R Peters S Mok T Camidge DR Gadgeel SM Ou SI Circulating cell-free DNA as a prognostic biomarker in patients with advanced *ALK*+ non-small cell lung cancer in the global phase III ALEX trial. Clin Cancer Res. 2022;28:1800–8. 10.1158/1078-0432.CCR-21-2840 35275991 PMC9365376

[20] Knapp B Mezquita L Devarakonda S Aldea M Waqar SN Pepin K Exploring the feasibility of utilizing limited gene panel circulating tumor DNA clearance as a biomarker in patients with locally advanced non-small cell lung cancer. Front Oncol. 2022;12:856132. 10.3389/fonc.2022.856132 35419282 PMC9000093

[21] Palmero R Taus A Viteri S Majem M Carcereny E Garde-Noguera J Biomarker discovery and outcomes for comprehensive cell-free circulating tumor DNA *versus* standard-of-care tissue testing in advanced non-small-cell lung cancer. JCO Precis Oncol. 2021;5:93–102. 10.1200/PO.20.00241 34994593

[22] Schouten RD Vessies DCL Bosch LJW Barlo NP van Lindert ASR Cillessen SAGM Clinical utility of plasma-based comprehensive molecular profiling in advanced non-small-cell lung cancer. JCO Precis Oncol. 2021;5:PO.20.00450. 10.1200/PO.20.00450 34632253 PMC8277301

[23] Fiala O Baxa J Svaton M Benesova L Ptackova R Halkova T Combination of circulating tumour DNA and 18F-FDG PET/CT for precision monitoring of therapy response in patients with advanced non-small cell lung cancer: a prospective study. Cancer Genomics Proteomics. 2022;19:270–81. 10.21873/cgp.20319 35181593 PMC8865037

[24] Shi Y Lei Y Liu L Zhang S Wang W Zhao J Integration of comprehensive genomic profiling, tumor mutational burden, and PD-L1 expression to identify novel biomarkers of immunotherapy in non-small cell lung cancer. Cancer Med. 2021;10:2216–31. 10.1002/cam4.3649 33655698 PMC7982619

[25] Cheng ML Lau CJ Milan MSD Supplee JG Riess JW Bradbury PA Plasma ctDNA response is an early marker of treatment effect in advanced NSCLC. JCO Precis Oncol. 2021;5:PO.20.00419. 10.1200/PO.20.00419 34250387 PMC8232122

[26] Kowanetz M Zou W Shames DS Cummings C Rizvi N Spira A OA20.01 Tumor mutation burden (TMB) is associated with improved efficacy of atezolizumab in 1L and 2L+ NSCLC patients. J Thorac Oncol. 2017;12:S321–2. 10.1016/j.jtho.2016.11.343

[27] Ross JS Goldberg ME Albacker LA Gay LM Agarwala V Elvin JA Immune checkpoint inhibitor (ICPI) efficacy and resistance detected by comprehensive genomic profiling (CGP) in non-small cell lung cancer (NSCLC). Ann Oncol. 2017;28:v404. 10.1093/annonc/mdx376.004

[28] Singal G Miller PG Agarwala V Li G Gossai A Albacker LA Analyzing biomarkers of cancer immunotherapy (CIT) response using a real-world clinico-genomic database. Ann Oncol. 2017;28:v404–5. 10.1093/annonc/mdx376.005

[29] Kang SP Gergich K Lubiniecki GM de Alwis DP Chen C Tice MAB Pembrolizumab KEYNOTE-001: an adaptive study leading to accelerated approval for two indications and a companion diagnostic. Ann Oncol. 2017;28:1388–98. 10.1093/annonc/mdx076 30052728 PMC5452070

[30] Hellmann MD Ciuleanu TE Pluzanski A Lee JS Otterson GA Audigier-Valette C Nivolumab plus ipilimumab in lung cancer with a high tumor mutational burden. N Engl J Med. 2018;378:2093–104. 10.1056/NEJMoa1801946 29658845 PMC7193684

[31] Rittmeyer A Barlesi F Waterkamp D Park K Ciardiello F von Pawel J et al.; OAK Study Group. Atezolizumab *versus* docetaxel in patients with previously treated non-small-cell lung cancer (OAK): a phase 3, open-label, multicentre randomised controlled trial. Lancet. 2017;389:255–65. Erratum in: Lancet. 2017;389:e5. 10.1016/S0140-6736(16)32517-X 27979383 PMC6886121

[32] Popat S Hubner R Houlston RS. Systematic review of microsatellite instability and colorectal cancer prognosis. J Clin Oncol. 2005;23:609–18. 10.1200/JCO.2005.01.086 15659508

[33] Hause RJ Pritchard CC Shendure J Salipante SJ. Classification and characterization of microsatellite instability across 18 cancer types. Nat Med. 2016;22:1342–50. Erratum in: Nat Med. 2017;23:1241. Erratum in: Nat Med. 2018;24:525. 10.1038/nm1017-1241a 27694933

[34] Yang G Zheng RY Jin ZS. Correlations between microsatellite instability and the biological behaviour of tumours. J Cancer Res Clin Oncol. 2019;145:2891–9. 10.1007/s00432-019-03053-4 31617076 PMC6861542

[35] Yanagawa N Yamada N Sugimoto R Osakabe M Uesugi N Shiono S The frequency of DNA mismatch repair deficiency is very low in surgically resected lung carcinoma. Front Oncol. 2021;11:752005. 10.3389/fonc.2021.752005 34692533 PMC8527876

[36] De Marchi P Berardinelli GN Cavagna RO Pinto IA da Silva FAF Duval da Silva V Microsatellite instability is rare in the admixed brazilian population of non-small cell lung cancer: a cohort of 526 cases. Pathobiology. 2022;89:101–6. 10.1159/000520023 34781284

[37] Brown LC Tucker MD Sedhom R Schwartz EB Zhu J Kao C LRP1B mutations are associated with favorable outcomes to immune checkpoint inhibitors across multiple cancer types. J Immunother Cancer. 2021;9:e001792. 10.1136/jitc-2020-001792 33653800 PMC7929846

[38] Cabrita R Lauss M Sanna A Donia M Skaarup Larsen M Mitra S Tertiary lymphoid structures improve immunotherapy and survival in melanoma. Nature. 2020;577:561–5. Erratum in: Nature. 2020;580:E1. 10.1038/s41586-020-2155-6 31942071

[39] Helmink BA Reddy SM Gao J Zhang S Basar R Thakur R B cells and tertiary lymphoid structures promote immunotherapy response. Nature. 2020;577:549–55. 10.1038/s41586-019-1922-8 31942075 PMC8762581

[40] Si H Kuziora M Quinn KJ Helman E Ye J Liu F A blood-based assay for assessment of tumor mutational burden in first-line metastatic NSCLC treatment: results from the MYSTIC study. Clin Cancer Res. 2021;27:1631–40. 10.1158/1078-0432.CCR-20-3771 33355200

[41] Wang Y Tong Z Zhang W Zhang W Buzdin A Mu X FDA-approved and emerging next generation predictive biomarkers for immune checkpoint inhibitors in cancer patients. Front Oncol. 2021;11:683419. 10.3389/fonc.2021.683419 34164344 PMC8216110

[42] Nabet BY Esfahani MS Moding EJ Hamilton EG Chabon JJ Rizvi H Noninvasive early identification of therapeutic benefit from immune checkpoint inhibition. Cell. 2020;183:363–76.e13. 10.1016/j.cell.2020.09.001 33007267 PMC7572899

[43] Weber S van der Leest P Donker HC Schlange T Timens W Tamminga M Dynamic changes of circulating tumor DNA predict clinical outcome in patients with advanced non-small-cell lung cancer treated with immune checkpoint inhibitors. JCO Precis Oncol. 2021;5:1540–53. Erratum in: JCO Precis Oncol. 2022;6:e2100566. 10.1200/PO.21.00182 34994642

[44] Xiong A Nie W Zhou Y Li C Gu K Zhang D Comutations in DDR pathways predict atezolizumab response in non-small cell lung cancer patients. Front Immunol. 2021;12:708558. 10.3389/fimmu.2021.708558 34630387 PMC8499805

[45] Lu J Zhang Y Lou Y Yan B Zou B Hu M ctDNA-profiling-based UBL biologicalprocess mutation status as a predictor of Atezolizumab response among TP53-negative NSCLC patients. Front Genet. 2021;12:723670. 10.3389/fgene.2021.723670 34557222 PMC8452871

[46] Bettegowda C Sausen M Leary RJ Kinde I Wang Y Agrawal N Detection of circulating tumor DNA in early- and late-stage human malignancies. Sci Transl Med. 2014;6:224ra24. 10.1126/scitranslmed.3007094 24553385 PMC4017867

[47] Hubers AJ Heideman DAM Yatabe Y Wood MD Tull J Tarón M EGFR mutation analysis in sputum of lung cancer patients: a multitechnique study. Lung Cancer. 2013;82:38–43. 10.1016/j.lungcan.2013.07.011 23927883

[48] Morikawa K Kinoshita K Kida H Inoue T Mineshita M. Preliminary results of NGS gene panel test using NSCLC sputum cytology and therapeutic effect using corresponding molecular-targeted drugs. Genes (Basel). 2022;13:812. 10.3390/genes13050812 35627198 PMC9141607

[49] Roncarati R Lupini L Miotto E Saccenti E Mascetti S Morandi L Molecular testing on bronchial washings for the diagnosis and predictive assessment of lung cancer. Mol Oncol. 2020;14:2163–75. 10.1002/1878-0261.12713 32441866 PMC7463327

[50] Zhai J Han S Guo Q Shan B Wang J Guo Y Identifying genomic alterations in small cell lung cancer using the liquid biopsy of bronchial washing fluid. Front Oncol. 2021;11:647216. 10.3389/fonc.2021.647216 33987084 PMC8110515

[51] Zhang X Li C Ye M Hu Q Hu J Gong Z Bronchial washing fluid *versus* plasma and bronchoscopy biopsy samples for detecting epidermal growth factor receptor mutation status in lung cancer. Front Oncol. 2021;11:602402. 10.3389/fonc.2021.602402 33828971 PMC8020887

[52] Tu HY Li YS Bai XY Sun YL Zheng MY Ke EE Genetic profiling of cell-free DNA from pleural effusion in advanced lung cancer as a surrogate for tumor tissue and revealed additional clinical actionable targets. Clin Lung Cancer. 2022;23:135–42. 10.1016/j.cllc.2021.09.002 34645582

[53] Chaudhuri AA Chabon JJ Lovejoy AF Newman AM Stehr H Azad TD Early detection of molecular residual disease in localized lung cancer by circulating tumor DNA profiling. Cancer Discov. 2017;7:1394–403. 10.1158/2159-8290.CD-17-0716 28899864 PMC5895851

[54] Qiu B Guo W Zhang F Lv F Ji Y Peng Y Dynamic recurrence risk and adjuvant chemotherapy benefit prediction by ctDNA in resected NSCLC. Nat Commun. 2021;12:6770. 10.1038/s41467-021-27022-z 34799585 PMC8605017

[55] Gale D Heider K Ruiz-Valdepenas A Hackinger S Perry M Marsico G Residual ctDNA after treatment predicts early relapse in patients with early-stage non-small cell lung cancer. Ann Oncol. 2022;33:500–10. 10.1016/j.annonc.2022.02.007 35306155 PMC9067454

